# The epidemiology of benzodiazepine-related toxicity in Ontario, Canada: a population-based descriptive study

**DOI:** 10.17269/s41997-023-00784-3

**Published:** 2023-06-15

**Authors:** Tonya J. Campbell, Siyu Men, Dana Shearer, Terry Ebejer, Matt Joosse, Josephine Quercia, Jane Sanders, Mina Tadrous, Tony Antoniou, Tara Gomes, on behalf of the Ontario Drug Policy Research Network Citizens’ Panel

**Affiliations:** 1 Li Ka Shing Knowledge Institute of St. Michael’s Hospital, 30 Bond St., Toronto, ON M5B 1W8 Canada; 2 ICES, Toronto, ON Canada; 3 Ontario Drug Policy Research Network Citizens’ Panel, St. Michael’s Hospital, Toronto, ON Canada; 4 Leslie Dan Faculty of Pharmacy, University of Toronto, Toronto, ON Canada; 5 Women’s College Hospital, Toronto, ON Canada; 6 Department of Family and Community Medicine, University of Toronto, Toronto, ON Canada; 7 Department of Family and Community Medicine, St. Michael’s Hospital, Toronto, ON Canada; 8 Institute of Health Policy, Management & Evaluation, University of Toronto, Toronto, ON Canada

**Keywords:** Benzodiazepines, Drug overdose, Controlled substances, Adolescent, Young adult, Health services, Benzodiazépines, mauvais usage des médicaments prescrits, substances réglementées, adolescent, jeune adulte, services de santé

## Abstract

**Objectives:**

Despite the widespread use of prescription benzodiazepines, there are few studies examining trends and patterns of benzodiazepine-related toxicity. We describe the epidemiology of benzodiazepine-related toxicity in Ontario, Canada.

**Methods:**

We conducted a population-based, cross-sectional study of Ontario residents who had an emergency department visit or hospitalization for benzodiazepine-related toxicity between January 1, 2013 and December 31, 2020. We reported annual crude and age-standardized rates of benzodiazepine-related toxicity overall, by age, and by sex. In each year, we characterized the history of benzodiazepine and opioid prescribing among people who experienced benzodiazepine-related toxicity, and reported the percentage of encounters with opioid, alcohol, or stimulant co-involvement.

**Results:**

Between 2013 and 2020, there were 32,674 benzodiazepine-related toxicity encounters among 25,979 Ontarians. During this period, the crude rate of benzodiazepine-related toxicity declined overall, from 28.0 to 26.1 per 100,000 population (age-standardized rate: 27.8 to 26.4 per 100,000), but increased among young adults aged 19 to 24 (39.9 to 66.6 per 100,000 population). Moreover, by 2020, the percentage of encounters associated with active benzodiazepine prescriptions had declined to 48.9%, while the percentage of encounters that had opioid, stimulant, or alcohol co-involvement rose to 28.8%.

**Conclusion:**

Benzodiazepine-related toxicity has declined in Ontario overall, but has increased among youth and young adults. Furthermore, there is growing co-involvement of opioids, stimulants, and alcohol, which may reflect the recent emergence of benzodiazepines in the unregulated drug supply. Multifaceted public health initiatives comprising harm reduction, mental health supports, and promotion of appropriate prescribing are needed to reduce benzodiazepine-related harm.

**Supplementary Information:**

The online version contains supplementary material available at 10.17269/s41997-023-00784-3.

## Résumé

**Objectifs:**

Malgré l’utilisation généralisée des benzodiazépines sur ordonnance, peu d’études portent sur les tendances et les schémas de toxicité de ces médicaments. Nous décrivons l’épidémiologie de la toxicité liée aux benzodiazépines en Ontario, au Canada.

**Méthode:**

Nous avons mené une étude populationnelle transversale des résidentes et résidents de l’Ontario ayant visité le service des urgences ou été hospitalisés pour toxicité liée aux benzodiazépines entre le 1^er^ janvier 2013 et le 31 décembre 2020. Nous avons rapporté globalement, par âge et par sexe les taux annuels de toxicité liée aux benzodiazépines, bruts et standardisés pour l’âge. Pour chaque année, nous avons caractérisé les antécédents de prescription de benzodiazépines et d’opioïdes chez les personnes ayant présenté une toxicité liée aux benzodiazépines, et rapporté le pourcentage de rencontres présentant une co-implication avec les opioïdes, l’alcool ou les stimulants.

**Résultats:**

Entre 2013 et 2020, il y a eu 32 674 rencontres pour toxicité liée aux benzodiazépines avec 25 979 Ontariens et Ontariennes. Durant cette période, le taux brut de toxicité liée aux benzodiazépines a baissé dans l’ensemble, passant de 28 à 26,1 pour 100 000 habitants (taux standardisé pour l’âge : 27,8 à 26,4 p. 100 000), mais il a augmenté chez les jeunes adultes de 19 à 24 ans (de 39,9 à 66,6 p. 100 000). De plus, en 2020, le pourcentage de rencontres associées à des ordonnances actives de benzodiazépines avait baissé à 48,9 %, tandis que le pourcentage de rencontres présentant une co-implication avec les opioïdes, les stimulants ou l’alcool avait augmenté à 28,8 %.

**Conclusion:**

La toxicité liée aux benzodiazépines a diminué en Ontario dans l’ensemble, mais elle a augmenté chez les jeunes et les jeunes adultes. De plus, cette toxicité présente une co-implication croissante avec les opioïdes, les stimulants ou l’alcool, ce qui peut refléter l’émergence récente des benzodiazépines dans l’approvisionnement non réglementé en drogues. Des initiatives de santé publique multidimensionnelles incluant la réduction des méfaits, le soutien en santé mentale et la promotion de la prescription appropriée sont nécessaires pour réduire les méfaits liés aux benzodiazépines.

## Introduction

Benzodiazepines are commonly used for the treatment of anxiety disorders, insomnia, and seizure disorders, as well as for alcohol withdrawal, muscle relaxation, and sedation (Hirschtritt et al., 2021; Longo & Johnson, 2000). Due in part to the large number of indications, the prevalence of benzodiazepine use is high, ranging between 5% and 10% in Canada and the United States between 1996 and 2019 (Agarwal & Landon, 2019; Alessi-Severini et al., 2014; Ontario Drug Policy Research Network on behalf of the ODPRN Citizens’ Panel, 2021; Weir et al., 2018). Notably, over the past two decades, several Western countries have also reported increases in the prevalence of benzodiazepine prescribing among children, youth, and adults less than 30 years of age (Huerta et al., 2016; Ontario Drug Policy Research Network on behalf of the ODPRN Citizens’ Panel, 2021; Sidorchuk et al., 2018).

In contrast to studies describing the prevalence of benzodiazepine use, and likely owing to the favourable safety profile of pharmaceutical benzodiazepines (Kang et al., 2022), there is a relative lack of research on trends in benzodiazepine-related toxicity. Sometimes referred to as ‘benzodiazepine overdose’ or ‘poisoning’, benzodiazepine toxicity can occur when these drugs are taken in high doses or when they are ingested with other central nervous system depressants, such as alcohol or opioids. People experiencing benzodiazepine-related toxicity typically present with central nervous system depression, including drowsiness, ataxia, slurred speech, altered mental status, and in severe cases, a comatose state. Respiratory depression may also occur, particularly when benzodiazepines are taken with other central nervous system depressants (Kang et al., 2022; Longo & Johnson, 2000). Notably, a recent US study reported significant increases in non-fatal and fatal benzodiazepine toxicity incidents, both with and without opioid involvement, between 2019 and 2020 (Liu et al., 2021). This increase coincides with the recent emergence of non-pharmaceutical benzodiazepines in the unregulated drug supply in both the USA and Canada, which has amplified the risk of benzodiazepine-related harm, particularly among people who use drugs (Canadian Centre on Substance Use & Addiction, 2021). However, the study only covered a two-year period, which highlights the need for comprehensive, population-based research examining long-term trends and patterns of benzodiazepine-related toxicity.

Accordingly, we sought to describe the epidemiology of benzodiazepine-related toxicity in Ontario, Canada. Our specific objectives were to study trends over time in the rate of healthcare encounters for benzodiazepine-related toxicity, characteristics of individuals who experienced benzodiazepine-related toxicity, and trends in prescribing history and polysubstance involvement in benzodiazepine-related toxicity incidents.

## Methods

### Study design and setting

We conducted a population-based, repeated cross-sectional study of healthcare encounters for benzodiazepine-related toxicity among residents of Ontario, Canada, between January 1, 2013 and December 31, 2020.

### Data sources

We obtained data from ICES (formerly known as the Institute for Clinical Evaluative Sciences), an independent, non-profit research institute whose legal status under Ontario’s health information privacy law allows it to collect and analyze health care and demographic data (ICES, 2023b). For Ontario residents, physician and hospital services are covered through the publicly funded Ontario Health Insurance Plan (OHIP), and records from these encounters are stored in databases held at ICES (ICES, 2023a, 2023c).

We used the Canadian Institute for Health Information’s National Ambulatory Care Reporting System and Discharge Abstract Database to identify emergency department visits and inpatient hospitalizations, respectively. We used the OHIP Registered Persons Database to determine the population of Ontario eligible for the provincial health insurance plan and to ascertain the demographic and vital status characteristics of the study population. We obtained records for dispensed benzodiazepine and opioid prescriptions from the Narcotics Monitoring System, which captures all prescriptions for controlled substances dispensed from community pharmacies in Ontario, regardless of payer. We identified outpatient care visits using the OHIP Claims database. These datasets were linked using unique encoded identifiers and analyzed at ICES. The use of the data in this project is authorized under Sect. 45 of Ontario’s Personal Health Information Protection Act and does not require review by a Research Ethics Board.

### Study population

We identified all emergency department visits and hospitalizations where benzodiazepine toxicity was recorded as the main problem, most responsible diagnosis, or as a secondary diagnosis using the *International Statistical Classification of Diseases and Related Health Problems, Tenth Revision, Canada* (ICD-10-CA) diagnosis code T42.4 (poisoning by benzodiazepines). We did not include emergency department visits or hospitalizations that were scheduled or in which the diagnosis was recorded as uncertain. We linked episodes of care in which patients were transferred between emergency departments, from an emergency department to inpatient care, or between facilities for inpatient care. Encounters were then defined as one unique episode of care for benzodiazepine-related toxicity. To allow for data linkage, we restricted our analysis to encounters among Ontario residents. We further excluded incidents among residents of long-term care homes, as we aimed to assess patterns of benzodiazepine-related toxicity in a community setting. For each individual who had a benzodiazepine toxicity encounter, we defined the index date as the date of their first encounter for benzodiazepine toxicity during the study period.

### Characteristics of people who experienced a benzodiazepine-related toxicity

For each person who experienced a benzodiazepine toxicity encounter during the study period, we measured their age, sex (due to limitations in our data source, we were unable to measure gender), location of residence (urban vs. rural; northern vs. southern), and neighbourhood-level income quintile on the index date (i.e., their first encounter for benzodiazepine toxicity during the study period). Geographic variables were derived using the Postal Code^OM^ Conversion File Plus and were determined based on the postal code on the individual’s health card. Rural communities were defined as those with a population less than 10,000, and all other communities were defined as urban (Statistics Canada, 2019). Northern Ontario was defined as the North East and North West Local Health Integration Networks. We also captured prior healthcare encounters for benzodiazepine-related toxicity, opioid-related toxicity, alcohol use disorder, and mental health and substance use disorders (see Table S1 in Supplementary Material for diagnosis codes) in the one year before the index date. To assess polysubstance involvement, we identified encounters for benzodiazepine-related toxicity which also included a diagnostic code for opioid-, alcohol-, or stimulant-related toxicity (Table S1). Finally, we measured the number of benzodiazepine toxicity encounters that each individual experienced over the study period (categorized as 1, 2, or 3 or more). In order to examine how characteristics differed by benzodiazepine prescription status, they were stratified according to whether or not an individual had an active benzodiazepine prescription at the time of the index toxicity encounter, defined as having a benzodiazepine dispensed on or prior to the encounter date with a day’s supply overlapping that date.

### Trends

We calculated annual crude and age-standardized (to the 2021 Ontario population) rates of benzodiazepine-related toxicity encounters per 100,000 Ontario residents overall and by sex. We also measured age-specific rates of benzodiazepine toxicity across the study period (0–18, 19–24, 25–34, 35–44, 45–64, 65–74, and 75 + years). In each year of the study period, we examined the percentage of encounters in which the individual had an active (defined above) or recent (dispensed in the 30 and 180 days prior to the encounter) benzodiazepine prescription, an active opioid prescription, and active prescriptions for both a benzodiazepine and an opioid. Finally, to investigate trends in polysubstance involvement in benzodiazepine toxicity incidents, we determined the annual percentage of encounters in which a diagnosis code for opioid-, alcohol-, or stimulant-related toxicity was recorded in conjunction with the benzodiazepine toxicity code. In these analyses, we considered all encounters for benzodiazepine toxicity over the study period. If an individual had multiple encounters for benzodiazepine toxicity during this period, all were included.

### Statistical analysis

We used descriptive statistics to summarize the characteristics of individuals who experienced benzodiazepine-related toxicity, overall and stratified by whether the individual had an active benzodiazepine prescription on the index date. We calculated standardized differences to compare the characteristics of people with and without an active benzodiazepine prescription, with differences greater than 0.10 considered meaningful (Mamdani et al., 2005). Poisson regression was used to estimate the annual percent change (APC) in the crude rates of benzodiazepine toxicity and the associated 95% confidence interval. We used the Cochran-Armitage test for trend to identify temporal changes in polysubstance involvement in benzodiazepine toxicity incidents and in the percentage of encounters in which the individual had active or recent benzodiazepine and opioid prescriptions. All analyses were conducted at ICES using SAS Enterprise Guide, version 7.1 (SAS Institute, Inc., Cary, NC) and used a type 1 error rate of 0.05 as the threshold for statistical significance.

### Citizen involvement

We regularly engage a Citizens’ Panel, consisting of up to 25 volunteer citizens from across Ontario, to help identify research areas of interest and ensure that citizen perspectives are incorporated throughout the research process. The Citizens’ Panel identified the topic of benzodiazepine toxicity as being of interest to policymakers, researchers, and the public. We engaged with the entire Citizens’ Panel, and a sub-committee of four representatives from the Panel who are co-authors on this study, to select the research question and measures, and to obtain feedback on the presentation and interpretation of study findings.

## Results

Between January 1, 2013 and December 31, 2020, there were 32,939 benzodiazepine-related toxicity encounters in Ontario. We excluded 56 encounters (0.2%) among people who resided outside of Ontario, and 209 encounters (0.6%) among individuals who resided in long-term care homes in Ontario. The final analytic dataset comprised 32,674 benzodiazepine-related toxicity encounters among 25,979 individuals (Figure S1).

### Characteristics of people who experienced benzodiazepine-related toxicity

Approximately two thirds of individuals (60.3%; N = 15,658) who experienced benzodiazepine-related toxicity over the study period were female, and the median age was 38 years (interquartile range [IQR] 24 to 53 years) (Table 1). Approximately half (51.7%; N = 13,422) resided in neighbourhoods in the two lowest quintiles of neighbourhood income. Notably, in the one year prior to the index benzodiazepine toxicity, 45.9% of people (N = 11,935) were admitted to an emergency department or hospital for a mental health or substance use disorder. During the same period, 5.6% of individuals (N = 1466) had a prior benzodiazepine toxicity encounter, 4.3% (N = 1105) had a previous opioid toxicity incident, and 16.2% (N = 4198) had a prior encounter for alcohol use disorder. Polysubstance involvement was common, with one quarter of individuals (24.8%; N = 6447) also having an opioid-, alcohol-, or stimulant-related toxicity recorded on the index encounter, of which opioid toxicity was most common (14.9%; N = 3858). Overall, 15.0% of people (N = 3900) experienced more than one benzodiazepine toxicity encounter over the study period. Relative to people who did not have an active benzodiazepine prescription on the index date, people with an active benzodiazepine prescription on index (52.8%; N = 13,715) tended to be older, had a higher prevalence of mental health and substance use disorders (including alcohol use disorder), were less likely to have co-occurring stimulant involvement, and were more likely to experience subsequent benzodiazepine-related toxicity incidents over the study period (Table 1).

**Table 1:** Characteristics of people who experienced a benzodiazepine-related toxicity from 2013 to 2020, overall, and by benzodiazepine prescription status

	Overall	Active benzodiazepine prescription	No active benzodiazepine prescription
Number of individuals	25,979	13,715	12,264
Age, years			
Median (IQR)	38 (24–53)	45 (30–56)*	30 (20–47)
0–18	3105 (12.0%)	633 (4.6%)*	2472 (20.2%)
19–24	3700 (14.2%)	1487 (10.8%)*	2213 (18.0%)
25–34	4628 (17.8%)	2346 (17.1%)	2282 (18.6%)
35–44	4075 (15.7%)	2333 (17.0%)	1742 (14.2%)
45–64	7954 (30.6%)	5203 (37.9%)*	2751 (22.4%)
65–74	1466 (5.6%)	1026 (7.5%)*	440 (3.6%)
75 +	1051 (4.0%)	687 (5.0%)	364 (3.0%)
Sex			
Female	15,658 (60.3%)	8586 (62.6%)	7072 (57.7%)
Male	10,321 (39.7%)	5129 (37.4%)	5192 (42.3%)
Location of residence			
Rural	2584 (9.9%)	1339 (9.8%)	1245 (10.2%)
Urban	23,239 (89.5%)	12,304 (89.7%)	10,935 (89.2%)
Missing	156 (0.6%)	72 (0.5%)	84 (0.7%)
Residence in northern Ontario	2189 (8.4%)	1144 (8.3%)	1045 (8.5%)
Neighbourhood income quintile			
1 (lowest)	7766 (29.9%)	4281 (31.2%)	3485 (28.4%)
2	5656 (21.8%)	3084 (22.5%)	2572 (21.0%)
3	4575 (17.6%)	2388 (17.4%)	2187 (17.8%)
4	3967 (15.3%)	1980 (14.4%)	1987 (16.2%)
5 (highest)	3826 (14.7%)	1900 (13.9%)	1926 (15.7%)
Missing	189 (0.7%)	82 (0.6%)	107 (0.9%)
Emergency department visit or hospital admission for benzodiazepine toxicity (past year)	1466 (5.6%)	857 (6.2%)	609 (5.0%)
Emergency department visit or hospital admission for opioid toxicity (past year)	1105 (4.3%)	605 (4.4%)	500 (4.1%)
Outpatient visit, emergency department visit, or hospital admission for alcohol use disorder (past year)	4198 (16.2%)	2538 (18.5%)*	1660 (13.5%)
Emergency department visit or hospital admission for mental health or substance use disorders (past year)	11,935 (45.9%)	6995 (51.0%)*	4940 (40.3%)
Anxiety disorders	5006 (19.3%)	3068 (22.4%)*	1938 (15.8%)
Deliberate self-harm	3554 (13.7%)	2041 (14.9%)	1513 (12.3%)
Mood disorders	4909 (18.9%)	3089 (22.5%)*	1820 (14.8%)
Schizophrenia	1224 (4.7%)	783 (5.7%)	441 (3.6%)
Substance-related disorders	4130 (15.9%)	2344 (17.1%)	1786 (14.6%)
Other	1848 (7.1%)	1099 (8.0%)	749 (6.1%)
Any opioid, alcohol, or stimulant toxicity in the episode of care for benzodiazepine toxicity	6447 (24.8%)	3238 (23.6%)	3209 (26.2%)
Opioid toxicity	3858 (14.9%)	2041 (14.9%)	1817 (14.8%)
Alcohol toxicity	1988 (7.7%)	1050 (7.7%)	938 (7.6%)
Stimulant toxicity	1893 (7.3%)	654 (4.8%)*	1239 (10.1%)
Number of encounters for benzodiazepine-related toxicity over the study period			
1	22,079 (85.0%)	11,153 (81.3%)*	10,926 (89.1%)
2	2666 (10.3%)	1671 (12.2%)*	995 (8.1%)
≥ 3	1234 (4.7%)	891 (6.5%)*	343 (2.8%)

Abbreviations: IQR – interquartile range

^*^Indicates a standardized difference > 0.10 when compared to individuals with no active benzodiazepine prescription

### Trends in benzodiazepine-related toxicity

**Fig. 1:**
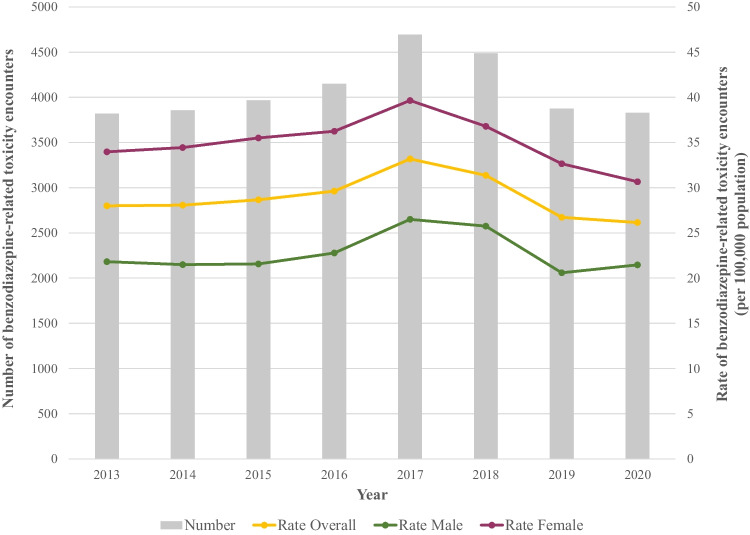
Trends in the number and crude rate of healthcare encounters for benzodiazepine-related toxicity, overall and by sex, 2013 to 2020

**Fig. 2:**
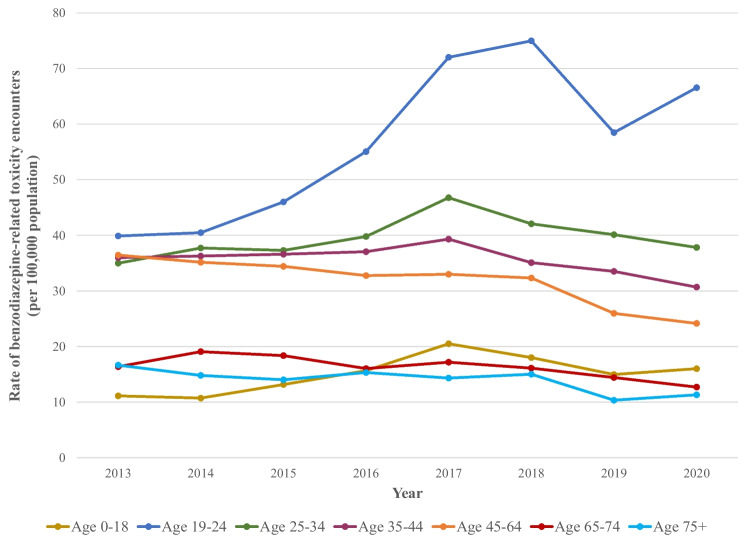
Age-specific trends in healthcare encounters for benzodiazepine-related toxicity, 2013 to 2020

The crude rate of benzodiazepine-related toxicity in Ontario rose from 28.0 per 100,000 population (N = 3820) in 2013 to a peak of 33.2 per 100,000 population (N = 4693) in 2017, before declining to 26.1 per 100,000 population (N = 3828) in 2020 (APC -0.41, 95% confidence interval [CI] -0.88 to 0.07; Fig. 1; Table S2). Age-standardized rates showed similar trends (27.8 per 100,000 in 2013 to 26.4 per 100,000 in 2020; Table S3). Crude rates were higher, but also showed a significant decline, among females (34.0 per 100,000 in 2013 to 30.7 per 100,000 in 2020; APC -0.89, 95% CI -1.49 to -0.29) compared to males, for whom rates remained stable (21.8 per 100,000 in 2013 to 21.5 per 100,000 in 2020; APC 0.38, 95% CI -0.38 to 1.15; Fig. 1; Table S2), and these trends were consistent after age adjustment (Table S3). Rates significantly increased among youth aged 18 or below (11.1 per 100,000 in 2013 to 16.0 per 100,000 in 2020; APC 6.09, 95% CI 4.56 to 7.64), young adults aged 19 to 24 (39.9 per 100,000 in 2013 to 66.6 per 100,000 in 2020; APC 8.38, 95% CI 7.03 to 9.73), and adults aged 25 to 34 (35.0 per 100,000 in 2013 to 37.8 per 100,000 in 2020; APC 1.45, 95% CI 0.34 to 2.56; Fig. 2; Table S2). Notably, for these age groups, although the rate of benzodiazepine-related toxicity increased between 2013 and 2020 overall, the rates reached a peak in 2017 before declining slightly between 2018 and 2020. In contrast, rates of benzodiazepine-related toxicity significantly decreased among people aged 35 and above, with the largest reduction observed in the 45 to 64 age group (36.4 per 100,000 in 2013 to 24.1 per 100,000 in 2020; APC -5.1, 95% CI -5.91 to -4.28). Importantly, by 2020, the rate of benzodiazepine-related toxicity among young adults aged 19 to 24 was two to six times higher than that observed in other age groups (66.6 per 100,000 vs. range of 11.3 to 37.8 per 100,000 in all other age groups).

### Prescription history prior to benzodiazepine-related toxicity

The percentage of benzodiazepine toxicity encounters in which the individual had an active benzodiazepine prescription declined from 61.1% (N = 2332) in 2013 to 48.9% (N = 1872) in 2020 (p < 0.0001; Table 2). Similar declining trends were observed in the dispensing of any benzodiazepine prescription in the 30 and 180 days prior to the toxicity encounter. There were also comparable reductions in the percentage of encounters in which the individual had an active opioid prescription (19.3% in 2013 to 14.1% in 2020; p < 0.0001), and active concomitant opioid and benzodiazepine prescriptions (15.7% in 2013 to 10.2% in 2020; p < 0.0001).

**Table 2:** Prevalence of dispensed benzodiazepine and opioid prescriptions among people with healthcare encounters for benzodiazepine-related toxicity, 2013 to 2020

Measure	2013	2014	2015	2016	2017	2018	2019	2020	p-value
Number of benzodiazepine-related toxicity encounters	3820	3856	3967	4149	4693	4487	3874	3828	
Benzodiazepine prescribing history									
Active prescription	2332 (61.1%)	2419 (62.7%)	2384 (60.1%)	2400 (57.9%)	2445 (52.1%)	2434 (54.3%)	2086 (53.9%)	1872 (48.9%)	< 0.0001
Prescription in the previous 30 days	2417 (63.3%)	2475 (64.2%)	2492 (62.8%)	2525 (60.9%)	2621 (55.9%)	2566 (57.2%)	2259 (58.3%)	2045 (53.4%)	< 0.0001
Prescription in the previous 180 days	2945 (77.1%)	3033 (78.7%)	3085 (77.8%)	3123 (75.3%)	3302 (70.4%)	3241 (72.2%)	2843 (73.4%)	2571 (67.2%)	< 0.0001
Active opioid prescription	738 (19.3%)	714 (18.5%)	741 (18.7%)	662 (16.0%)	716 (15.3%)	671 (15.0%)	580 (15.0%)	539 (14.1%)	< 0.0001
Active opioid and benzodiazepine prescriptions	600 (15.7%)	569 (14.8%)	577 (14.5%)	500 (12.1%)	532 (11.3%)	507 (11.3%)	435 (11.2%)	391 (10.2%)	< 0.0001

### Polysubstance involvement in benzodiazepine-related toxicity

Polysubstance (i.e., opioid, alcohol or stimulant) involvement in benzodiazepine toxicity incidents increased from 22.1% (N = 844) in 2013 to 28.8% (N = 1101) in 2020 (p < 0.0001; Table 3). While the largest relative growth was a two-fold increase in stimulant involvement (4.9% in 2013 to 10.2% in 2020; p < 0.0001), opioid involvement in benzodiazepine toxicity incidents was most common throughout the study period (17.1% in 2020).

**Table 3:** Involvement of opioids, alcohol, and stimulants in healthcare encounters for benzodiazepine-related toxicity, 2013 to 2020

Measure	2013 (N = 3820)	2014 (N = 3856)	2015 (N = 3967)	2016 (N = 4149)	2017 (N = 4693)	2018 (N = 4487)	2019 (N = 3874)	2020 (N = 3828)	p-value
Any opioid, alcohol, or stimulant toxicity in the episode of care for benzodiazepine toxicity	844 (22.1%)	837 (21.7%)	971 (24.5%)	985 (23.7%)	1260 (26.9%)	1203 (26.8%)	981 (25.3%)	1101 (28.8%)	< 0.0001
Opioid toxicity	535 (14.0%)	483 (12.5%)	584 (14.7%)	555 (13.4%)	772 (16.5%)	696 (15.5%)	566 (14.6%)	653 (17.1%)	< 0.0001
Alcohol toxicity	253 (6.6%)	303 (7.9%)	331 (8.3%)	336 (8.1%)	352 (7.5%)	384 (8.6%)	299 (7.7%)	327 (8.5%)	< 0.05
Stimulant toxicity	187 (4.9%)	182 (4.7%)	198 (5.0%)	244 (5.9%)	396 (8.4%)	409 (9.1%)	324 (8.4%)	392 (10.2%)	< 0.0001

## Discussion

Between 2013 and 2020, the rate of benzodiazepine-related toxicity in Ontario, Canada significantly decreased among people aged 35 and older, and significantly increased among people under 35 years of age. Strikingly, by 2020, the rate among young adults aged 19 to 24 was at least double that of any other age group studied. Moreover, there was a decrease in the percentage of toxicity encounters in which the individual had an active benzodiazepine prescription, and there was an increase in incidents involving opioids, alcohol, and stimulants. Taken together, these trends suggest a growing contribution of non-pharmaceutical benzodiazepines in benzodiazepine-related toxicity incidents in Ontario.

In Ontario, the overall rate of benzodiazepine-related toxicity rose between 2013 and 2017, but showed a decline by 2020. This decline was particularly notable among encounters associated with active benzodiazepine prescriptions, which also showed a pronounced decrease starting between 2016 and 2017. This change may reflect the influence of US and Canadian guidelines, published in 2016 and 2017 respectively, cautioning against the combined use of benzodiazepines and opioids for chronic non-cancer pain (Busse et al., 2017; Dowell et al., 2016). Similar trends were observed in the rate of non-fatal emergency department visits for benzodiazepine-related toxicity in the USA between 2016 and 2017 (Vivolo-Kantor et al., 2020). However, more recent data from the USA suggests a reversal in these trends, as emergency department visits for benzodiazepine-related toxicity, both with and without opioid involvement, rose between 2019 and 2020 (Liu et al., 2021). This is in contrast to our findings, which demonstrated stability in the overall rate of benzodiazepine-related toxicity between 2019 and 2020. This difference may reflect the earlier emergence of non-approved ‘designer’ benzodiazepines (Marin & van Wijk, 2019) in the unregulated drug supply in the USA compared to Canada, as supported by the substantial increase in the seizure of these drugs, including etizolam, flualprazolam, and flubromazolam, in the USA between 2015 and 2019 (Bollinger et al., 2021). In contrast, data from drug checking services in Ontario began to show a rise in the detection of non-approved benzodiazepines in Toronto’s unregulated fentanyl supply during the early waves of the COVID-19 pandemic (Centre on Drug Policy Evaluation, 2022). Research describing patterns of opioid toxicity deaths in Ontario during the pandemic has also revealed an increasing involvement of non-approved benzodiazepines, as demonstrated by etizolam being detected in less than 5% of deaths in 2019, compared to 25% in 2020 (Gomes et al., 2021). This underscores the need for consistent monitoring of trends in benzodiazepine-related toxicity in Ontario, including sources of the benzodiazepines involved, both for early detection of changes in trends, and to inform public health initiatives to prevent benzodiazepine-related harm.

Across the study period, rates of benzodiazepine-related toxicity were highest among adults between the ages of 19 and 34. This is in line with findings of a recent US study demonstrating that rates of emergency department visits for non-medical benzodiazepine use between 2016 and 2017 were highest among teenagers and young adults (Moro et al., 2020). Importantly, our study advances the literature by examining long-term age-specific trends in benzodiazepine-related toxicity, which demonstrate that the rate of benzodiazepine toxicity has increased significantly between 2013 and 2020 among youth and young adults. It is notable that this mirrors trends in benzodiazepine prescribing observed in these age groups across several jurisdictions in Canada (Alessi-Severini et al., 2014; Ontario Drug Policy Research Network on behalf of the ODPRN Citizens’ Panel, 2021) and Europe (Huerta et al., 2016; Sidorchuk et al., 2018). However, future work is needed to elucidate whether these trends are being driven by the use of prescribed, diverted, or non-pharmaceutical benzodiazepines. Furthermore, a recent US study found that diagnoses related to mental health and substance use disorder were recorded on more than 50% of all emergency department visits for benzodiazepine toxicity among youth and young adults in 2016, and almost half of all incidents were documented as intentional (Bushnell et al., 2021). Although we did not limit our cohort to youth and young adults, we also observed a high prevalence of concurrent mental health and substance use disorder diagnoses, particularly anxiety and mood disorders, among people with and without an active benzodiazepine prescription on their index encounter. These findings demonstrate that efforts to prevent benzodiazepine-related harm, particularly among youth and young adults, should focus on strengthening and enhancing access to community-based mental health services and non-pharmacologic therapies.

We observed that the percentage of benzodiazepine toxicity encounters in which the individual had an active benzodiazepine prescription consistently decreased across the study period. At the same time, the percentage of encounters in which opioids, stimulants, or alcohol were also involved steadily increased, reaching almost 30% by 2020. The rising polysubstance involvement in benzodiazepine-related toxicity incidents is likely partially driven by increasing adulteration of the unregulated drug supply with non-approved benzodiazepines, which has been observed in Canada (British Columbia Coroners Service, 2022; Canadian Centre on Substance Use & Addiction, 2021; Gomes et al., 2021; Ontario Drug Policy Research Network et al., 2020) and the USA (Bollinger et al., 2021; Liu et al., 2021; Marin & van Wijk, 2019). However, it is important to note that although the contribution of prescription benzodiazepines to benzodiazepine-related toxicity encounters decreased over the study period, nearly 50% of encounters affected people with an active prescription, and more than two thirds affected those who had a prescription dispensed in the prior six months. Therefore, initiatives to prevent benzodiazepine-related harm should also focus on efforts to improve the safe use of prescription benzodiazepines.

### Limitations

Our study has several strengths, including the use of a comprehensive, population-based database to characterize long-term trends in benzodiazepine-related toxicity across the largest province in Canada. However, several limitations should be noted. First, we could not capture incidents that went untreated or were treated outside of a hospital setting. Furthermore, drug screening practices, including decisions around whether to screen and the drug classes included in screening, vary across emergency departments. As a result, some people presenting to the emergency department with benzodiazepine toxicity may have gone undiagnosed or misdiagnosed. Consequently, we have likely underestimated the true rate of benzodiazepine toxicity in Ontario. Second, our analysis of the involvement of opioids, stimulants, and alcohol relied on the use of diagnostic codes for toxicities related to these substances on the same record as the benzodiazepine encounter. However, some instances of polysubstance involvement may not be reported by patients or recorded on medical records, and some may go undetected, particularly in the emergency department setting. Third, because we did not have access to data on benzodiazepine toxicity deaths occurring outside of a hospital setting, we were unable to measure rates of fatal benzodiazepine toxicity. Although our study may include benzodiazepine toxicity encounters that had a fatal outcome in hospital, it does not explicitly identify encounters as such. Importantly, as non-approved benzodiazepines have become more common in the unregulated opioid supply, there may be an associated rise in benzodiazepine-related toxicity incidents that result in death before the patient reaches the hospital. As these incidents would not be captured by our study, it is possible that the reductions we observed in rates of benzodiazepine toxicity, particularly among middle-age groups, could partially be driven by a higher number of polysubstance-involved deaths occurring outside of hospital. Future studies should aim to examine trends in both fatal and non-fatal benzodiazepine toxicity events in Ontario using linked healthcare data and coronial records. Moreover, we did not quantify how characteristics and patterns of toxicity incidents differed by intent. Future work should examine how the intent of benzodiazepine-related toxicity differs across the age spectrum, in order to help inform and appropriately target initiatives related to prevention, harm reduction, and access to mental health support services. Finally, although we quantified the percentage of encounters among people with an active benzodiazepine prescription, we are not able to comment on whether toxicity incidents in this study were directly caused by prescribed, diverted, or non-pharmaceutical benzodiazepines.

## Conclusion

Although the rate of benzodiazepine-related toxicity has declined in Ontario, there has been a substantial increase in toxicity incidents among youth and young adults. Moreover, although the involvement of opioids, stimulants, and alcohol in benzodiazepine toxicity encounters is growing, almost 50% of encounters in 2020 affected people with an active benzodiazepine prescription. Therefore, in order to reduce benzodiazepine-related harm, there is a need for multifaceted public health approaches that focus on promoting the safe use of prescription benzodiazepines, improving access to harm reduction approaches such as drug checking services, providing safe alternatives to the unregulated drug supply, and strengthening and enhancing access to mental health services for youth and young adults.

## Contributions to knowledge

What does this study add to existing knowledge?

- This is the first study to characterize the epidemiology of benzodiazepine-related toxicity in Ontario.
- The overall rate of benzodiazepine-related toxicity has declined in Ontario. However, rates have increased substantially among youth and young adults, particularly those aged 19 to 24, among whom the rate in 2020 was at least double that of any other age group.
- There has been a decrease in the percentage of benzodiazepine toxicity encounters associated with prescription benzodiazepines, and simultaneously, polysubstance involvement in benzodiazepine-related toxicity incidents has grown. This may be reflective of the increasing adulteration of the unregulated drug supply with non-approved benzodiazepines.

What are the key implications for public health interventions, practice, or policy?

- Public health approaches to reducing benzodiazepine-related harm must be multifaceted, including policies and messaging to promote the safe and appropriate use of prescription benzodiazepines, strategies to improve access to harm reduction initiatives such as drug checking services for people using drugs from the unregulated supply, initiatives to provide safe alternatives to the unregulated drug supply, and efforts to strengthen and enhance access to community-based mental health services and non-pharmacologic therapies for youth and young adults.

### Supplementary Information

Below is the link to the electronic supplementary material.

Supplementary file1 (DOCX 32 kb)

## Data Availability

The dataset from this study is held securely in coded form at ICES. While legal data sharing agreements between ICES and data providers (e.g., healthcare organizations and government) prohibit ICES from making the dataset publicly available, access may be granted to those who meet pre-specified criteria for confidential access, available at www.ices.on.ca/DAS (email: das@ices.on.ca).

## Code availability

The full dataset creation plan and underlying analytic code are available from the authors upon request, understanding that the computer programs may rely upon coding templates or macros that are unique to ICES and are therefore either inaccessible or may require modification.
