# Supplementary material for: The epidemiology of benzodiazepine-related toxicity in Ontario, Canada: a population-based descriptive study
Source: Can J Public Health. 2023 Jun 15;114(6):956–66. doi: 10.17269/s41997-023-00784-3 (PMC10267543; doi:10.17269/s41997-023-00784-3)
Supplement: Supplementary file 1 — Supplementary file1 (DOCX 32 kb) [file 41997_2023_784_MOESM1_ESM.docx]

**Table S1.** Diagnostic codes and data sources used to define healthcare encounters

| **Measure** | **Data Source** | **Codes** |
| --- | --- | --- |
| **Benzodiazepine-related toxicity** | Emergency department visit (all diagnosis types), inpatient  hospitalization (all diagnosis  types) | **ICD-10-CA:** T42.4 |
| **Opioid-related toxicity** | **In the one year prior to benzodiazepine-related toxicity:** Emergency department visit (all diagnosis types), inpatient  hospitalization (admission diagnosis)  **At the time of benzodiazepine-related toxicity:** In the same episode of care as the benzodiazepine-related toxicity encounter | **ICD-10-CA:** T40.0, T40.1, T40.2, T40.3, T40.4, T40.6 |
| **Alcohol-related toxicity** | **In the one year prior to benzodiazepine-related toxicity:** Emergency department visit (all diagnosis types), inpatient  hospitalization (all diagnosis  types)  **At the time of benzodiazepine-related toxicity:** In the same episode of care as the benzodiazepine-related toxicity encounter | **ICD-10-CA:** T51.0 |
| **Stimulant-related toxicity** | **At the time of benzodiazepine-related toxicity:** In the same episode of care as the benzodiazepine-related toxicity encounter | **ICD-10-CA:** T43.6, T40.5 |
| **Alcohol use disorder** | Emergency department visit (all diagnosis types), inpatient  hospitalization (all diagnosis  types), or outpatient visit with a physician | **ICD-10-CA:** F10 Z50.2 Z71.4 Z864.0 Z72.1 K70 G31.2 G62.1 G72.1 I42.6 K29.2 K86.0 T51.0 E24.4 K85.2 |
|  |  | **OHIP DX:** 291, 303 |
|  |  | **DSM:** 291 303 30500 |
| **Anxiety disorders** | Emergency department visit (main problem), inpatient  hospitalization (most responsible diagnosis), or mental health hospitalization (primary discharge diagnosis, or provisional diagnosis if primary discharge diagnosis was missing) | **ICD-10-CA:** F40, F41, F42, F43, F48.8, F48.9, F93.1, F93.2 |
|  |  | **DSM:** 300.0, 300.2, 300.3, 308.3, 309.0, 309.24, 309.28, 309.3, 309.4, 309.8, 309.9  Provisional: 7, 15 |
| **Deliberate self-harm** | Emergency department visit (secondary problem field) or inpatient  hospitalization (secondary diagnosis) | **ICD-10-CA:** X60-X84, Y10-Y19, Y28 AND main problem or most responsible diagnosis NOT F06-F99 |
| **Mood disorders** | Emergency department visit (main problem), inpatient  hospitalization (most responsible diagnosis), or mental health hospitalization (primary discharge diagnosis, or provisional diagnosis if primary discharge diagnosis was missing) | **ICD-10-CA:** F30, F31, F32, F33, F34, F38, F39, F53.0 |
|  |  | **DSM:** 296, 300.4, 301.13, 311 Provisional: 6 |
| **Schizophrenia** | Emergency department visit (main problem), inpatient  hospitalization (most responsible diagnosis), or mental health hospitalization (primary discharge diagnosis, or provisional diagnosis if primary discharge diagnosis was missing) | **ICD-10-CA:** F20 (excluding F20.4), F22, F23, F24, F25, F28, F29, F53.1 |
|  |  | **DSM:** 295, 297, 298  Provisional: 5 |
| **Substance-related disorders** | Emergency department visit (main problem), inpatient  hospitalization (most responsible diagnosis), or mental health hospitalization (primary discharge diagnosis, or provisional diagnosis if primary discharge diagnosis was missing) | **ICD-10-CA:** F10, F11, F12, F13, F14, F15, F16, F17, F18, F19, F55 |
|  |  | **DSM**: 291 (excluding 291.82), 292 (excluding 292.85), 303, 304, 305  Provisional: 4 |
| **Other mental health disorders** | Emergency department visit (main problem), inpatient  hospitalization (most responsible diagnosis), or mental health hospitalization (primary discharge diagnosis, or provisional diagnosis if primary discharge diagnosis was missing) | **ICD-10-CA:** Any mental health disorder was defined as a main problem, most responsible diagnosis, or primary discharge diagnosis of F06-F99, OR secondary problem or diagnosis of X60-X84, Y10-Y19, Y28 when the main problem, most responsible diagnosis, or primary discharge diagnosis was NOT F06-F99.  Other mental health disorders were defined as anyone flagged with a mental health disorder as defined above, but not an indication of an anxiety disorder, deliberate self-harm, mood disorder, schizophrenia, or substance-related disorder. |
|  |  | **DSM**: Any mental health disorder was defined as any DSM code (excluding 290, 294, and a provisional diagnosis of 2 when the primary discharge diagnosis was missing).  Other mental health disorders were defined as anyone flagged with a mental health disorder as defined above, but not an indication of an anxiety disorder, deliberate self-harm, mood disorder, schizophrenia, or substance-related disorder. |

**Abbreviations:** DSM – Diagnostic and Statistical Manual of Mental Disorders; ICD-10-CA – International Classification of Diseases, 10th Revision, Canada; OHIP DX – Ontario Health Insurance Plan diagnostic code

**Fig S1.** Flowchart illustrating the process of identifying records for the analytic dataset

**32,674**

Encounters (unique episodes of care) for benzodiazepine-related toxicity among
**25,979** community-dwelling Ontarians

**32,939**

Encounters (unique episodes of care) for benzodiazepine-related toxicity in Ontario between
January 1, 2013 and December 31, 2020

**56** (0.2%) encounters among people residing outside of Ontario

**209** (0.6%) encounters among people residing in long-term care homes

**Table S2.** Trends in the number and crude rate of healthcare encounters for benzodiazepine-related toxicity, overall, by age, and by sex, 2013 to 2020^*^

| **Year** | **2013** | **2014** | **2015** | **2016** | **2017** | **2018** | **2019** | **2020** | **APC (95% CI)** |
| --- | --- | --- | --- | --- | --- | --- | --- | --- | --- |
| **Overall** | 3,820 (28.0) | 3,856 (28.1) | 3,967 (28.7) | 4,149 (29.6) | 4,693 (33.2) | 4,487 (31.4) | 3,874 (26.7) | 3,828 (26.1) | -0.41 (-0.88, 0.07) |
| **Females** | 2,356 (34.0) | 2,405 (34.4) | 2,499 (35.5) | 2,580 (36.3) | 2,850 (39.7) | 2,675 (36.8) | 2,406 (32.7) | 2,283 (30.7) | **-0.89 (-1.49, -0.29)** |
| **Males** | 1,464 (21.8) | 1,451 (21.5) | 1,468 (21.6) | 1,569 (22.8) | 1,843 (26.5) | 1,812 (25.8) | 1,468 (20.6) | 1,545 (21.5) | 0.38 (-0.38, 1.15) |
| **Age 0-18** | 325  (11.1) | 312  (10.7) | 382  (13.2) | 456  (15.7) | 599  (20.5) | 529  (18.0) | 442  (15.0) | 474  (16.0) | **6.09 (4.56, 7.64)** |
| **Age 19-24** | 431  (39.9) | 438  (40.5) | 495  (46.0) | 589  (55.1) | 764  (72.0) | 789  (75.0) | 614  (58.5) | 688  (66.6) | **8.38 (7.03, 9.73)** |
| **Age 25-34** | 635  (35.0) | 691  (37.7) | 694  (37.3) | 758  (39.8) | 907  (46.8) | 835  (42.1) | 818  (40.1) | 789  (37.8) | **1.45 (0.34, 2.56)** |
| **Age 35-44** | 674  (36.0) | 673  (36.3) | 675  (36.6) | 683  (37.0) | 728  (39.3) | 658  (35.1) | 640  (33.5) | 595  (30.7) | **-1.82 (-2.96, -0.67)** |
| **Age 45-64** | 1,413 (36.4) | 1,373 (35.2) | 1,355 (34.4) | 1,301 (32.8) | 1,316 (33.0) | 1,292 (32.3) | 1,038 (26.0) | 964  (24.1) | **-5.1 (-5.91, -4.28)** |
| **Age 65-74** | 187  (16.4) | 228  (19.1) | 229  (18.4) | 208  (16.0) | 231  (17.2) | 224  (16.1) | 208  (14.4) | 190  (12.7) | **-4.1 (-6.07, -2.09)** |
| **Age 75+** | 155  (16.6) | 141  (14.8) | 137  (14.0) | 154  (15.3) | 148  (14.3) | 160  (15.0) | 114  (10.3) | 128  (11.3) | **-4.88 (-7.26, -2.44)** |

**Abbreviations:** APC – annual percent change; CI – confidence interval.

Boldface indicates a statistically significant change in annual percent change at α = 0.05.

*****Data shown are the number (crude rate per 100,000 population) of healthcare encounters for benzodiazepine-related toxicity in Ontario.

**Table S3.** Trends in the age-standardized rate of healthcare encounters for benzodiazepine-related toxicity, overall and by sex, 2013 to 2020^*^

| **Year** | **Overall** | **Females** | **Males** |
| --- | --- | --- | --- |
| **2013** | 27.8 | 33.5 | 21.8 |
| **2014** | 28.0 | 34.2 | 21.5 |
| **2015** | 28.5 | 35.2 | 21.6 |
| **2016** | 29.6 | 36.1 | 22.8 |
| **2017** | 33.3 | 39.6 | 26.7 |
| **2018** | 31.5 | 36.9 | 26.0 |
| **2019** | 26.9 | 32.8 | 20.8 |
| **2020** | 26.4 | 30.9 | 21.7 |

*****Data shown are the age-standardized rate (per 100,000 population) of healthcare encounters for benzodiazepine-related toxicity in Ontario. Rates were age-standardized to the 2021 Ontario population using the direct method.
